# Assessment of the face validity, feasibility and utility of a patient-completed questionnaire for polymyalgia rheumatica: a postal survey using the QQ-10 questionnaire

**DOI:** 10.1186/s40814-017-0150-y

**Published:** 2017-07-06

**Authors:** Helen Twohig, Georgina Jones, Sarah Mackie, Christian Mallen, Caroline Mitchell

**Affiliations:** 1 Academic Unit of Primary Medical Care, University of Sheffield, Samuel Fox House, Northern General Hospital, Herries Road, Sheffield, S5 7AU UK; 2 Faculty of Health and Social Sciences, Leeds Beckett University, Leeds, UK; 3 Leeds Institute of Rheumatic and Musculoskeletal Medicine, University of Leeds, Leeds, UK; 4 Arthritis Research UK Primary Care Centre, Institute for Primary Care and Health Sciences, Keele University, Keele, UK; 5 Academic Unit of Primary Medical Care, University of Sheffield, Sheffield, UK

**Keywords:** Polymyalgia rheumatica, Patient perspective, Outcomes research, Patient-reported outcome measures, Questionnaire validity and utility assessment

## Abstract

**Background:**

The development of a patient-reported outcome measure (PROM) for polymyalgia rheumatica (PMR), a condition that causes pain, stiffness and disability, is necessary as there is no current validated disease-specific measure. Initial literature synthesis and qualitative research established a conceptual framework for the condition along with a list of symptoms and effects of PMR that patients felt were important to them. These findings were used to derive the candidate items for a patient-completed questionnaire. We aim to establish the face validity of this initial “long form” of a PROM.

**Methods:**

People with a current or previous diagnosis of PMR were recruited both from the community and from rheumatology clinics. They were asked to complete the PMR questionnaire along with the QQ-10 questionnaire, which is a measure used to assess the face validity, feasibility and utility of patient healthcare questionnaires.

**Results:**

A total of 28 participants with an age range of 59–85 years and a length of time since diagnosis from 4 months to 18 years completed the QQ-10. The overall mean “value” score was 79% (SD 12), and the mean “burden” score was 21% (SD 18). The free-text comments were analysed thematically and were found to focus on layout, content, where in the clinical pathway the questionnaire would be most beneficial, specific items missing and other areas for consideration.

**Conclusions:**

The high mean value score and low burden score indicate that the questionnaire has good face validity and is acceptable to patients. The questionnaire now needs to undergo further psychometric evaluation and refinement to develop the final tool for use in clinical practice and research.

## Background

Polymyalgia rheumatica (PMR) causes significant pain, stiffness and disability in older adults and is treated with systemic glucocorticoids which can themselves cause significant additional morbidity [1, 2]. A recent systematic review of outcome measures used in PMR research studies [3] identified a wide variety of instruments in use, none of which were specifically developed to measure symptom burden in patients with PMR. Less than 10% of studies measured physical function, quality of life or fatigue, despite these being important to patients [4]. Further work, including a Delphi survey conducted by the OMERACT PMR Working Group, highlighted the need for a disease-specific outcome measure that would cover domains of life impact relevant to patients [5]. Applying international guidelines for patient-reported outcome measure (PROM) development [6], we carried out initial qualitative work with patients with PMR to better understand patient experience of the condition and establish a conceptual framework for a future PMR PROM [7]. Here, we report the next steps taken to derive a “long-list” of candidate items for a PMR-specific PROM and the assessment of face validity, feasibility and utility both in the participants of the original study and in a separate group of patients, using a validated method, the QQ-10 questionnaire [8]. The QQ-10 is a measure developed to collect standardised information on important aspects of a questionnaire’s qualities from the patient’s perspective and is used to assess the face validity, feasibility and utility of patient healthcare questionnaires. This part of the iterative PROM development process is particularly important to ensure that the instrument is acceptable and contains the content that is relevant to patients with PMR, and completion burden of the final questionnaire is minimised thus improving follow-up and questionnaire completion rates in clinical trials [9]. These data are vital for development of a PROM that is valid for either research studies or clinical practice.

## Methods

A long-list of candidate items for the PROM was developed from the data obtained from a previous qualitative study carried out by our group [7]. These items were categorised into main symptoms/duration (4 items), function (24 items), emotional and psychological well-being (11 items), steroid side effects (10 items) and overall well-being (1 item). Respondent validation of the themes and items was carried out with the original interview participants. All 20 interviewees from the qualitative study were sent a copy of the long-list and asked to return it with any comments that they had on the content. Ten of these individuals agreed to a structured telephone interview to discuss the long list in more detail and the information gathered from this was used to make changes to it to form the draft questionnaire (Table 1).

**Table 1:** Long-list of PMR PROM questionnaire items derived from the qualitative study

Item	Question
1	How severe has the pain from your PMR been in the last 2 weeks? (0-10 visual analogue scale (VAS) with 0 = no pain and 10 = the worst pain you’ve ever had)
2	How severe has the stiffness from your PMR been in the last 2 weeks? (0-10 VAS with 0 = no stiffness and 10 = the worst stiffness you’ve ever felt)
3	How severe has the weakness from your PMR been in the last 2 weeks? (0-10 VAS with 0 = no weakness and 10 = complete weakness)
4	On average, for how much of each day has the pain/stiffness/weakness from your PMR been present for during the last 2 weeks? All day/About half the day/Around 1-3 hours/< 1 hour
5	FUNCTION: Over the last 2 weeks, compared to what you can normally do, has PMR limited your ability to do the following activities? Graded as 1) no, not limited at all, 2) yes, limited a little, 3) yes, limited a lot, 4) not relevant Bend down Get up after bending down Get in and out of a car Drive a car Get in and out of bed Get in or out of a chair Get in or out of a bath Wash yourself fully Dry yourself fully after a shower/bath Take your coat on or off Put on or take off your socks and shoes Comb or blow dry your hair Get on or off the toilet Wipe yourself after going to the toilet Engage in intimate/sexual activity Walk up stairs Walk up hills Walk on the flat Carry or lift things Reach above your head for things Grip objects Do housework Do gardening Sit for more than 30 minutes at a time Participate in sports
6	EMOTIONAL AND PSYCHOLOGICAL WELL-BEING: In the last 2 weeks have your PMR symptoms… Graded as 1) none of the time, 2) a little of the time, 3) some of the time, 4) most of the time, 5) all of the time Caused you to feel low in mood Caused you to feel anxious Caused you to feel vulnerable Lowered your self-confidence Made you worried that you might fall over Caused you to need more help with looking after yourself Made you less inclined to go out Stopped you doing hobbies that you used to do Made you worry about the future Affected your sleep Made you feel more tired than usual
7	TREATMENT SIDE EFFECTS: How much have you been affected by side effects from your medication in the last 2 weeks? (VAS with 0 = unaffected, 10 = severely affected)
8	In the last 2 weeks, have you been bothered by any of the following side effects of your steroid medication? (Yes/No) Weight gain Change in appearance (fatter face, saggy skin) Irritability Low mood Euphoria Hyperactivity Easy bruising Indigestion Insomnia Hair loss
9	Do you feel back to the level of health you were at before you first experienced PMR symptoms? (Yes/No)

To assess face validity and utility in an independent group of patients, patients with a diagnosis of PMR were recruited through two routes: (1) community-based: through a patient-led patient support group, PMR&GCAUK North East Support and (2) hospital-based: through rheumatology clinics at Leeds Teaching Hospitals NHS Trust. This recruitment strategy was designed to sample from the full spectrum of patients with PMR as it is a condition managed in both primary and secondary care. Ethical approval was received from the National Institute for Social Care and Health Research Research Ethics Service, Wales REC 7 (Ref 12/WA/0344), and all participants provided informed consent. Patients received the study materials by post (community-based recruitment) or in a sealed envelope from their treating rheumatologist (hospital-based recruitment), completed the study materials at home in their own time, and returned the completed forms to the lead researcher (HT), who was based at a different institution and in a different city to their rheumatology clinic.

The data from the QQ-10 questionnaire were analysed both quantitatively and qualitatively. For quantitative analysis we used the QQ-10 scoring method [8]. Likert ratings from strongly disagree to strongly agree (coded as 0–4) were summed separately for the first six questions comprising the value score (helped me communicate about my condition, relevant to my condition, easy to complete, included all the aspects of my condition I am concerned about, was enjoyable, would be happy to complete as part of routine care), and from the last four questions comprising the burden score (too long, embarrassing, complicated, upset me).

Qualitative thematic analysis [10] was performed on comments received in response to the three free-text questions at the end of the QQ-10:

1. Do you have any comments or suggestions on how the questionnaire you used could be improved (e.g., its structure, appearance or design)?
2. Were any of your important symptoms, problems or concerns missed out by the questionnaire you used?
3. Do you feel that any areas or problems in the questionnaire you used were over-represented?

## Results

Twenty-eight patients took part (20 female and 8 male; age range 59–85 years; duration since PMR diagnosis 4 months to 18 years; apart from a single participant who was 18 years post diagnosis, no patient was more than 5 years post diagnosis). Eighteen of the participants were still on steroid treatment for their PMR.

**Fig. 1:**
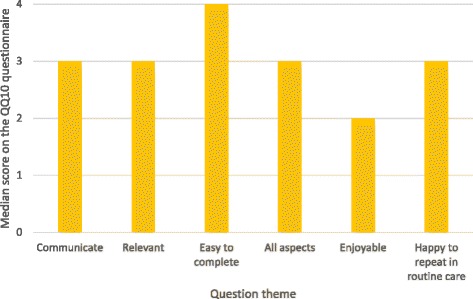
Mean value of domains contributing to the value score

**Fig. 2:**
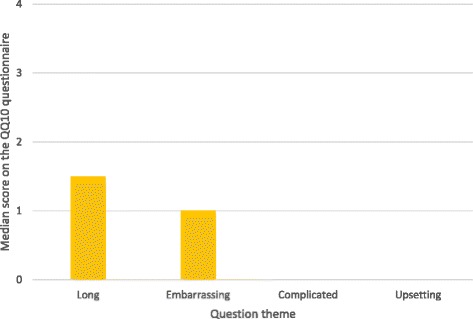
Mean value of domains contributing to the burden score

**Fig. 3:**
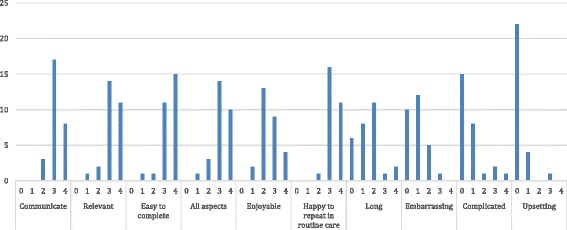
Frequency distribution of scores for each question in the QQ-10

The overall mean value score was 79% (SD 12), and the mean burden score was 21% (SD 18). The median of each domain making up the value score was >2 (range 0–4), and the median of each domain making up the burden score was <1.5 (range 0–4). Figures 1 and 2 show the median scores for each question, and Fig. 3 shows the spread of responses for each question.

The five emergent themes from the free-text comments (Table 2) were (1) layout, (2) content, (3) where in the clinical pathway the questionnaire would be most beneficial, (4) specific items not covered and (5) other areas for consideration for inclusion. The content theme encompassed sub-themes of depth and detail, specificity to PMR and heterogeneity of the condition.

**Table 2:** Thematic analysis of the QQ10 free-text answers

Theme	Sub-theme	Quote
Layout		“I think it’s well set out in a fairly simple and effective format.”
		“Where the table goes over the page, it would be helpful to repeat the headers. Page numbering would be helpful.”
		“Sheets should be numbered and column headings should be repeated where they go onto 2 pages.”
		“Questions about age etc. should be at the start.”
		“Figure diagrams are a more direct indication of the type and location of pain than the written word.”
Content	Depth and detail	“Too much detail, cut down on the number of boxes, they overlap too much. Q5 has too many choices.”
		“Seems quite straightforward.”
		“Q9 (Do you feel back to the level of health you were at before you had PMR?) - if answer is no, ask why?”
	Specificity to PMR	“PMR is not that specific. For me I sometimes ache and sometimes feel a bit down. Muscle power has diminished but that may be age.”
		“Many things you ask could be for other reasons such as depression, arthritis, cancer etc. it’s not all PMR.”
		“Some info on other conditions should be included e.g., I had had a stroke previously and PMR imposed symptoms on top of those resulting from that.”
		“Some symptoms (like weakness and difficulty reaching things in cupboards) I already had from a shoulder injury it’s difficult to tell whether the PMR made it worse or not.”
		“As everyone is different there should be room for an individual’s particular symptoms and concerns.”
		“Q9 is irrelevant, no-one gets back to feeling as well as before.”
	Heterogeneity	“Should pain/ache be quantified? Different people will have different meanings.”
		“Some questions didn’t seem to fit my symptoms but I don’t think I’m very severely affected - time will tell.”
		“PMR affects all of us in different ways and the questionnaire covers all aspects and does no harm even if some questions overlap.”
Where in the clinical pathway it would be most beneficial		“I think it’s an excellent questionnaire from the outset of a PMR diagnosis to a record of the PMR journey.”
		“I think the questionnaire will be very helpful, especially to people at the beginning of their treatment when they probably have all of the problems listed. I would have been reassured to think the doctors knew how I was feeling.”
Specific items missing		“The side effects of prednisolone”
		“Diabetes, fluid retention leading to lymphoedema”
		“The area of pain”
		“More questions about fatigue could be included. Skin/hair condition missed out.”
		“Swelling of the joints is not mentioned (hands, wrists, feet and ankles).”
		“No real questions about where the pain was or what I couldn’t do.”
		“One of my main concerns at the start was inability to fasten my own bra - had to ask for help.”
Other concerns that could be included		“Concerns about recurrence are important.”
		“You might like to know how the steroid treatment has helped/been successful.”
		“It may be helpful to ask patients to put down aspects of their health that may not be PMR related but which they wish to discuss.”

## Discussion

This study represents a distinct, patient-orientated phase within a stepped standardised methodological approach to developing a PROM for PMR for research and clinical practice. The use of the validated QQ-10 measure is an example of how to embed patient perspectives at all stages within the PROM development process, moving beyond the paradigm of clinician-orientated outcome measures.

The high value and low burden scores are encouraging as regards face validity and feasibility of the questionnaire. They are similar to scores obtained when the QQ-10 has been used in other studies, including evaluation of the King’s Health Questionnaire [11] and evaluation of use of a bladder diary [12], and both of these tools were judged as having been proven to be useful based on these results. The free-text comments provided added richness to the response data, and their analysis highlights some important required amendments to the structure and layout of the questionnaire as well as suggesting some additional points for inclusion. The comments related to content echo some of the findings from our earlier qualitative work [7] and some of the known challenges of outcome measurement in PMR, chiefly the heterogeneity of the condition, difficulty in assessment in the presence of co-morbidities and overlap with other conditions [1, 4, 13].

The strengths of this study include the use of a validated instrument (QQ-10) to assess face validity, feasibility and utility of instruments designed to assess the life impact of particular disease states. The QQ-10 itself was rigorously developed using standard psychometric methods and has been demonstrated to have high internal consistency and item correlation and to be acceptable and understandable to patients [8]. It also has the advantages of being quick and cheap to administer and allowing comparison of different versions of a measure at several stages during development. At this stage of questionnaire development, we made the decision not to employ interview-based qualitative methods, such as cognitive interviewing, as the postal method of responding did not exclude patients too frail to participate in cognitive interviews, and allowed patients to respond honestly, in private and without any concern that the responses might be seen by their treating clinicians. A further strength of this study was the use of a combination of recruitment methods which identified patients with a range of disease durations similar to that described in previous literature [14, 15].

The main limitation of this study was the small number of participants and opportunistic (and thus not statistically representative) sampling method. However, this sample size was appropriate for this stage of PROM development [16, 17]. A further limitation of this study was that the community-based method of recruitment required patients to self-identify as being diagnosed with PMR. We did not attempt to validate diagnosis by means of classification or other criteria designed to select patients with PMR for research studies, because we wanted to assess this questionnaire in a real-life setting for clinical practice, not just for use in research studies.

## Conclusions

The long form of our PMR questionnaire was shown to have face validity for patients and be acceptable for use in their care. The work reported here represents an essential step in the PROM development process, paving the way for further work using a larger sample size that will allow formal psychometric validation, item reduction and ultimately generation of a fully validated patient-reported outcome measure for PMR.

## Abbreviations

OMERACT: Outcome measures in rheumatology (www.omeract.org). An independent initiative of international health professionals interested in outcome measures in rheumatology
PMR: Polymyalgia rheumatica
PROM: Patient-reported outcome measure
